# Deciphering the binding strength of oil matrix through molecularly resolved release energy analysis using thermal slicing ramped pyrolysis GC-MS

**DOI:** 10.1007/s00216-025-06110-9

**Published:** 2025-10-10

**Authors:** Kaijun Lu, Jianhong Xue, Zhanfei Liu

**Affiliations:** 1 The University of Texas at Austin, Marine Science Institute, 750 Channel View Dr., Port Aransas, TX 78373 USA; 2 Department of Marine Science, Coastal Carolina University, 100 Chanticleer Drive East, Conway, SC 29526 USA

**Keywords:** Thermal slicing ramped pyrolysis, Release energy, Matrix strength, Petroleum-related organic matter

## Abstract

Recent advancements in analytical techniques have significantly furthered our understanding of the chemical composition of petroleum and its derivatives. However, there remains a missing link connecting the molecular structure with how specific compounds are bound within the sample matrix. Traditional approaches to study energetic characteristics of petroleum often rely on bulk property measurements (e.g., thermogravimetric analysis), therefore lacking the resolution needed to capture compound-specific interactions and structural information. In this work, we aim to fill the gap between compositional and energetic analyses by employing thermal slicing ramped pyrolysis gas chromatography mass spectrometry (TSRP-GC-MS). TSRP-GC-MS enables the analysis of temperature-dependent release patterns of pyrolyzates, linking molecular structures to energetic characteristics via computing the energy distributions for specific pyrolyzates. Results of the photodegradation experiments demonstrate that as the photodegradation proceeds, the energy required to disrupt the matrix and to release *n*-alkanes increased from ca. 90 kJ/mol to over 100 kJ/mol, with significant increases for each individual *n*-alkane. The release energy of individual *n*-alkanes in crude oil and in tarball collected in the field were further compared in the second case study. The results not only showed an increase from 110 kJ/mol for *n*-alkanes in crude oil to 118 kJ/mol in tarball, but also revealed a homogenizing trend of the matrix strength of different *n*-alkanes in the latter. Overall, the proposed TSRP-GC–MS approach offers a powerful tool for advancing our understanding of petroleum and oil and has the potential to be expanded to the study of other complex natural mixtures.

**Supplementary Information:**

The online version contains supplementary material available at 10.1007/s00216-025-06110-9.

## Introduction

Despite the maturity of the petroleum industry, significant advancements in analytical techniques for characterizing petroleum and its derivatives have still been achieved over the past decade [1]. The chemical structure of petroleum and its derivatives plays a crucial role in determining their physicochemical properties, chemical reactivity, and fate in the environment. Therefore, gaining better insights into the chemical structures on molecular level is one of the major objectives of the petrochemical research and oil spill science.

Given the complexity of petroleum and petroleum-related organic materials, a range of analytical techniques has been employed to unravel their intricate molecular structure, such as molecular formulas and functional groups. The integrated application of high-performance separation (e.g., high-performance liquid or gas chromatography), functional group analysis (e.g., infrared spectroscopy and nuclear magnetic resonance spectroscopy), high-resolution spectroscopy (e.g., mass spectrometry), and statistical data treatment has deepened our understanding of these organic materials [2]. For instance, the use of Fourier-transform ion cyclotron resonance mass spectrometry (FTICR-MS) has allowed a detailed profiling of the complex organic mixture, with the detection and assignment of many thousands of molecular formulas within one spectrum [3]. While ultrahigh-resolution MS may not be universally regarded as the “gold standard” for petroleum analysis [1, 3–5], results from FTICR-MS nevertheless have greatly advanced our understanding of the fate of crude oil after photooxidation in the environment, such as the detection of numerous oxygen (O) containing compounds (e.g., [6]).

In addition to compositional analysis, the “energetic characteristics” of petroleum and related materials has also gained considerable attention in geochemistry, environmental science, and industry [7, 8]. Unlike compositional studies, research on the energetic characteristics of oil focuses on understanding how specific fractions of petroleum and/or related organic materials undergo volatilization, decomposition, or transformation under different environmental or reaction conditions. The obtained results, generally in the form of energy distribution or specific kinetic parameters, can be further connected to the chemo- and bio-lability of the fraction. Therefore, insights into energetic characteristics are crucial for predicting the environmental fate of petroleum (e.g., transport, degradation) and optimizing industrial processes such as refining.

Despite the advancements over the past decades, there remains a missing link in connecting the molecular level information on oil-related organic matter with its energetic behavior. While compositional analyses reveal what compounds are present, they do not explain how those compounds differ in their release, transformation, or degradation. Specifically, from the perspective of geochemistry, it remains unclear under what conditions different molecules in oil are released from the sample matrix and how the preservation of these molecules relates to their molecular structures. Traditional approaches to investigating energetic characteristics of oil often rely on bulk property measurements, such as changes in weight, density, viscosity, or boiling point (e.g., [1, 9]). Techniques like thermogravimetric analysis (TGA) and ramped pyrolysis oxidation (RPO) have been widely used to evaluate the thermal oxidation behavior of crude oil (e.g., [10, 11]), demonstrating that the weathering process shifts the thermograms to higher temperature regions (i.e., more CO_2_ produced in higher temperature region in more degraded petroleum samples [11]). These measurements are less demanding in terms of analytical power, but they inevitably omit the molecular-level details of the petroleum sample. In addition, these “low-resolution” techniques could potentially suffer from “intrinsic averaging” [2], leading to similar results between rather different samples. Essentially, the energetic characteristics of oil and its derivatives depend on molecular-scale interactions and transformations, such as bond breaking, cracking, or oxidation, which vary widely among individual compounds. Yet, bulk properties cannot capture the specific energies for different reaction pathways, the chemical diversity (e.g., aliphatics vs. aromatics), or the functional group chemistry (e.g., alkanes vs. unsaturated vs. aromatics). Without this resolution, models based on bulk properties often struggle in differentiating between samples with different molecular compositions but similar macroscopic properties.

Bridging this gap requires analytical approaches that integrate molecular-level characterization with energy analysis. One promising approach is to couple the characterization tools (e.g., MS) with a reactor (e.g., pyrolyzer, photoionization reactor), in which specific groups of compounds can be isolated and/or specific intermediate products can be identified and quantified. Among these reactors, pyrolyzer has been one of the most commonly used reactors (e.g., [12–14]). Classic pyrolysis GC-MS provides insights into the products generated during rapid or flash thermal decomposition (e.g., from room temperature to over 600 °C within a few seconds), but it cannot offer sufficient information on the precise temperatures at which specific compounds or pyrolyzates are released [12, 13, 15, 16]. Therefore, there is a need to have a “temperature resolution” that can capture the temperature-dependent release patterns of pyrolyzates and link them to molecular structures [17, 18].

In this work, we introduced a combination of thermal slicing ramped pyrolysis gas chromatography mass spectrometry (TSRP-GC-MS). Similar ramped pyrolysis techniques (either with thermal slicing or with double-shot) have already been applied in petrochemistry to achieve a temperature-resolved molecular characterization of oil-related samples, including plastics (e.g., [15, 19–21]), but we further explored its application in energetic analysis to quantify the energy required to release individual hydrocarbons from the sample matrix on a molecular level. Unlike RPO, TSRP-GC-MS operates under anoxic conditions, allowing the molecular identity of released pyrolyzates to be preserved. This difference also means that the energy derived from this analysis are fundamentally different from the “activation energy” reported for oxic thermal decomposition (e.g., [22]). TSRP-GC-MS is similar to the principle of using multiple cold traps pyrolysis [17, 18], except that a sample can be programmed in and out of a furnace to control the temperature resolution of the pyrolysis.

The estimated energy via this pyrolysis-based technique can be further separated into two different categories based on the pyrolysis temperature or if cracking is present. In the relatively low temperature range (the non-cracking zone), the measured energy reflects both the energy required to release the molecule from its surrounding matrix (i.e., the disruption of intermolecular interactions with other molecules) and the energy needed for volatilization (i.e., the enthalpy of vaporization). The former term is therefore further defined as the matrix strength in this work. At higher temperatures (the cracking zone), energy needed for breaking covalent bond is also included. With the pyrolyzates produced in different temperature intervals (i.e., “thermal slices”) being captured and analyzed by the GC-MS, temperature-dependent release patterns of specific pyrolyzate categories can be further transformed into energy distributions, offering a novel means of connecting molecular structures to energetic behavior and reactivity [23].

Here, we demonstrated the approach of using data from TSRP-GC-MS to quantify the energetic behavior of oil-related samples with two cases studies: one is the analysis of the photodegradation products of crude oil, and the other one is the comparison of crude oil and tarball samples. Based on assumption that the release of intact individual alkanes from an oil sample is a first-order, independent reactions [24], these applications illustrate the potential of this method to enhance our understanding of the molecular-level release energy of oil-related organic matter and the binding strength of individual hydrocarbons in an oil matrix.

## Materials and procedures

### Sample analyses

Photo-generated asphaltene samples were acquired from a photooxidation experiment described in a previous study [25]. Briefly, the pre-weathered light Louisiana sweet crude oil (180 °C overnight) was mixed with seawater to a final concentration of 1000 ppm, and the slurry was incubated under natural light and dark condition (Port Aransas, Texas) for up to 44 days. The asphaltene fraction of the incubation samples was isolated via the liquid-liquid extraction (i.e., SARA fractionation) at designated time points (days 0, 5, and 44).

To compare the energetic behavior of crude oil and tarballs, reference oil SRM 2779 from the Macondo wellhead was obtained from the National Institute of Science and Technology (NIST) and is referred to as “crude oil” herein [19]. The tarball samples spanning a time range of 881 days after the wellhead blowout of Deepwater Horizon (DWH) oil spill (April 10, 2010) were collected from the Grand Isle or within Bay Jimmy, Louisiana (29.258°N 89.958°W and 29.477°N 89.894°W, respectively [19, 26]). Of all the collected tarball samples, the 337 days post spill tarball (“337d tarball”) was selected for TSRP-GC-MS analysis and subsequent activation energy calculation.

*n*-Alkane standards for the identification of produced pyrolyzates were purchased from Sigma-Aldrich [19, 27].

### Thermal slicing ramped pyrolysis gas chromatography mass spectrometry (TSRP-GC-MS)

**Fig. 1:**
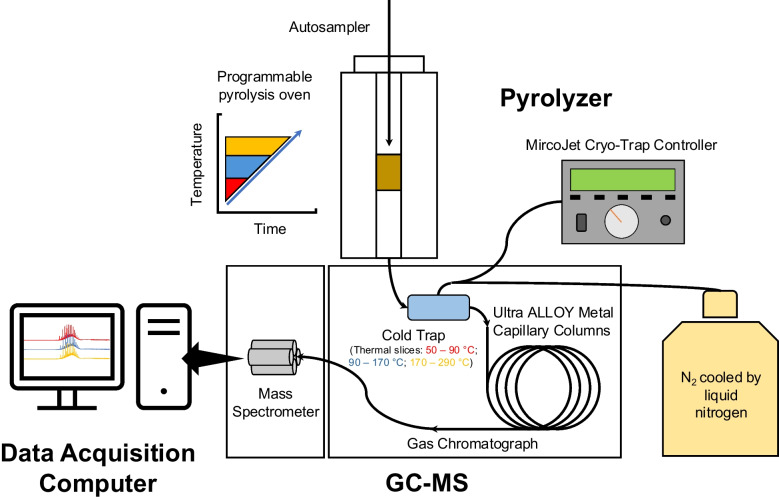
A schematic figure of the TSRP-GC-MS system. Different thermal slices and their corresponding chromatograms are represented by different colors (red, blue, and yellow) for illustration purpose

A schematic figure of the instrumentation is demonstrated in Figure 1. Protocols for thermal slicing ramped pyrolysis GC-MS (TSRP-GC-MS) have been described in our previous works [19, 28, 29]. Briefly, oil samples (with an equivalent amount of at least 1 mg of C) were analyzed through a multi-shot pyrolyzer (EGA/PY-3030D, Frontier Laboratories Ltd.) coupled with GC-MS (Shimadzu GCMS-TQ8040). The pyrolysis was conducted under helium, and the pyrolyzer was set to ramp from 50 to 650 °C at a constant heating rate of 20 °C·min^−1^ following previous work (e.g., [30]). Up to six consecutive thermal slices were selected: 50–90 °C, 90–170 °C, 170–290 °C, 290–370 °C, 370–530 °C, and 530–650 °C. These thermal slices were chosen based on our previous work (e.g., [19, 28, 29]), as they can provide ample separation of different compound classes within both natural organic matter samples and petroleum-related samples. These slices can be further categorized into different zones: dehydration (50–170 °C), dehydration and decarboxylation (170–290 °C), depolymerization (290–370 °C), and fragmentation and cracking (370–650 °C) [19, 25, 30, 31].

Within each thermal slice, as the temperature rose in the pyrolyzer, the produced pyrolyzates were condensed in a cold trap, which was held at −190 °C by the N_2_ gas connected to liquid N_2_. The cold temperature from the cold trap prevents the pyrolyzates from passing through the GC column. Once the pyrolyzer reached the preset temperature of the thermal slice, the cold trap was turned off to release the condensed pyrolyzates to the GC column (Figure 1). The pyrolyzates were analyzed following a standard GC-MS workflow with helium as the carrier gas. The GC oven was set at 40 °C and held for 2 min, heated at 10 °C·min^−1^ to 150 °C and then at 20 °C·min^−1^ to 320 °C, and held for 3 min. The pyrolyzates were ionized via electron ionization (EI). The mass spectrometer was programed to detect ions in the range of 26–600 mass-over-charge ratios (*m/z*). Compared with previous techniques, such as multiple cold trap pyrolysis [17, 18], TSRP-GC-MS allows the trapped pyrolyzates to undergo standard GC separation once released. This offers additional molecular structural information and facilitates the characterization processes of the pyrolyzates.

Molecule identification was achieved through comparison with either purchased standards or NIST open-source databases provided through the GC-MS software package. Given the focus on the *n*-alkane of the oil samples, a level one identification of *n*-alkane-like pyrolyzates was achieved through a match in the GC retention time and a match in the MS spectrum with the commercial standards [32]. However, for more complex natural organic samples where standards may not be available, the assignments of the pyrolyzates are relied on the comparison of spectra with the database (e.g., [28, 29]).

Pyrolysis of organic matter is an extremely complicated process that involves many secondary reactions, especially at high temperature zones [33]. Given the goal of this work is to decipher the energy required to volatilize intact compounds (i.e., the matrix strength), the subsequent analysis is focused more on the low temperate zones (50–370 °C) where less secondary reactions and fragmentations occur. In other words, compounds from the lower temperature zones should be more representative of the original sample matrix than those from higher temperature zones.

### Construction of the thermogram

**Fig. 2:**
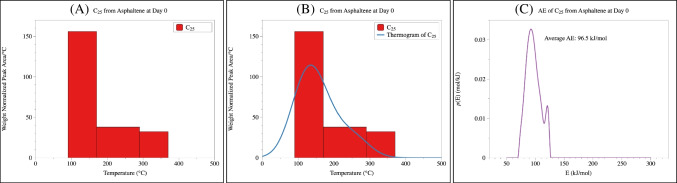
A typical piecewise bar graph showing the intensity of pyrolyzates C_25 _*n*-alkane (**A**), which can be transformed to a continuous distribution based on kernel density estimation (**B**) and can be subsequently computed to generate the activation energy distribution (**C**)

A typical TSRP-GC-MS dataset, as shown in Table 1, can be transformed into piecewise distribution representing the derivative thermogravimetric curve (DTG) of the organic material (Figure 2A). This step can either be applied to the total pyrolyzates or modified to target specific categories of pyrolyzates (e.g., Figure 2A). In the piecewise distribution, a constant production rate of pyrolyzates (i.e., intensity per degree) is assumed within a specific thermal slice. For instance, the production rate of *n*-alkane C_25_ is calculated to be 155.9 °C^−1^ for the slice of 90–170 °C (Table 1) using the equation of

$$\text{Production rate}=\left(\frac{12473.1}{170-90}\right)=155.9 {^\circ \text{C}}^{-1}$$

**Table 1:** Weight normalized intensities for selected *n*-alkanes from the isolated asphaltene at day 0 [25]

Day 0 asphaltene	50–90 °C	90–170 °C	170–290 °C	290–370 °C	370–530 °C	530–650 °C
C14	2929.8	0.0	0.0	0.0	5384.9	0.0
C15	11,608.7	1646.8	0.0	0.0	4827.6	0.0
C16	13,983.3	8628.5	0.0	0.0	4215.1	0.0
C17	9468.3	20,539.1	0.0	0.0	3974.7	0.0
C18	2680.4	29,991.3	0.0	0.0	3318.9	0.0
C19	0.0	34,543.6	670.8	0.0	3268.6	0.0
C20	0.0	37,886.9	496.2	0.0	3133.3	0.0
C21	0.0	35,225.3	432.7	0.0	2447.4	0.0
C22	0.0	29,987.5	748.7	0.0	2731.4	0.0
C23	0.0	24,233.7	1539.7	0.0	2033.3	0.0
C24	0.0	18,587.8	2787.2	0.0	2434.9	0.0
C25	0.0	12,473.1	4580.1	2588.8	2661.9	0.0
C26	0.0	5837.5	5562.5	1800.0	2300.6	0.0
C27	0.0	3695.5	6430.1	1818.3	2269.9	0.0
C28	0.0	1725.3	5209.3	713.8	1353.5	0.0
C29	0.0	780.1	6674.7	933.7	1412.2	0.0

This piecewise distribution is then smoothed to a continuous distribution using the kernel density estimation (KDE) function in the “stats” package in R (RStudio version 2023.06.1+524, Figure 2B, R script file in Supporting Information). The continuous distribution takes the form of the superposition of several normal distributions, and the width of each normal distribution is specifically adjusted so that the integration matches that of the original piecewise distribution over the selected temperature range (i.e., the integrated total intensity over temperature is the same).

Such transformation is built upon a basic assumption in distributed activation energy model (DAEM), which states that the pyrolytic decomposition of an organic material is carried out through a large number of independent reactions corresponding to different “pseudo-components” [24]. The global pyrolytic decomposition behavior then reflects the individual behavior of these pseudo-components, which can be further depicted by a Gaussian distribution. For instance, the pyrolysis of lignocellulosic biomass, such as corn stover, cotton stalk, and palm oil husk, can be successfully described as the sum of three independent components (i.e., hemicellulose, cellulose, and lignin) following Gaussian distributions [24, 34–36]. The constructed DTG, which is based on evolved gas analysis (EGA) data, also agrees well with the DTG curve obtained from thermogravimetric analysis (TGA) (Figure S1) conducted under similar conditions (same temperature range to 650 °C, anoxic condition under N_2_), indicating that the transformation is valid.

### The calculation of release energy of pyrolyzates

The concept of an activation energy distribution was originally proposed by Vand [37] to investigate how the electrical resistance of metallic films changes and was later applied to the study of the kinetics of volatile products from coal by Pitt [38] and Anthony and Howard [39]. DAEM has now been widely applied in the pyrolysis field to probe the relationship between the thermal activation energy of organic matter and its weight change or the production of volatile matters (e.g., [24, 40, 41]). Note that in this work, even though the conventional DAEM terminology of “activation energy” is used in describing the method, the calculated energy (especially in the low temperature regions) represents the energy required to release specific pyrolyzates from the matrix.

The deducing processes as well as the application of DAEM have been thoroughly presented and discussed in many studies (e.g., [34, 35, 42]) and can also be found in the Supplementary Material. Briefly, in the continuous DAEM, the pyrolysis process of an organic matter can be viewed as an infinite number of irreversible first order parallel reactions with continuously varying activation energies occurring simultaneously. Therefore, the amount of volatile material at a given condition is a function of the energy (*E*), and the conversion factor *α* that describes how much of the total amount of volatile products (V*) has already been released by time *t* can be expressed as

1 $$\alpha =\frac{V}{{V}^{*}}=1-{\int }_{0}^{\infty }\text{exp}\left[-{\int }_{{T}_{0}}^{T}\frac{A}{\beta }\text{exp}\left(-\frac{E}{RT}\right)\text{d}T\right]f\left(E\right)\text{d}E=1-{\int }_{0}^{\infty }\Phi \left(E,T\right)f\left(E\right)\text{d}E$$

in which *E* is the specific activation energy level, *f(E)* is a distribution function that describes the probability for the chemical groups to have an activation energy of *E*,* A* is the empirically derived Arrhenius pre-exponential factor, *R* is the ideal gas constant, *T* is the measured temperature at time *t*, *T*_*0*_ is the starting temperature, and *β* is the ramping rate, given a linear heating process*.* This classic equation of *α* has been frequently mentioned in different forms in the field of thermal analyses (e.g., [34, 35, 42–44]). During a thermogravimetric or pyrolysis process, the ramping rate *β* is often preset for a specific heating profile, and *α* is generally monitored as the amount of generated volatile products, or the amount of remaining mass of a sample (i.e., 1 − *α*). As the frequency factor *A* is also interrelated with *E* and *f(E)* (e.g., [43–45]), knowing how *α* changes with *T* at a given *β* theoretically helps to calculate the *f(E)* and understand the distribution function of activation energy.

Previous studies have taken various approaches to simplify Eq. (1) and to solve *A* and *f(E)*; however, a perfect approach is yet to be found [34, 42–44, 46–48]. The most current approach to estimate the distribution function *f(E)* is provided by Hemingway et al. [42]. The core of this work was to use the inverse solution to calculate the *f(E)* that provides the best match of the calculated distribution of *α* vs. *T* with the observed distribution, with *A* being set as a priori (i.e., a constant of 10^10^ s^−1^). A Tikhonov regularization factor *λ* is further introduced to minimize the issue of noises in measured data and to avoid an overfit. The extensive calculation is achieved with Python code and is accessible through the “rampedpyrox” package [42].

### Modification of the approach

In this work, the inverse solution is applied with modifications. Specifically, the production of CO_2_ in the original code (i.e., equivalent to 1 – *α* in Eq. (1)) is substituted with the DTG data based on peak intensities (“Construction of the thermogram” section). The activation energy distribution and the averaged activation energy are calculated in Python (version 3.11.7, Figure 2C). The major difference between RPO and TSRP-GC-MS is the introduction of O_2_ into the pyrolysis process in the former technique, which leads to a complete transformation of organic C into CO_2_ with negligible amount of residual left in RPO [42]. In contrast, the TSRP-GC-MS analysis preserves the volatilized pyrolyzates, which are subsequently separated and identified via GC-MS, explicitly retaining the molecular-level information of the complex oil samples.

Moreover, due to the introduction of O_2_, the estimated activation energy from RPO may be more representative of the reactions between organic matters and O_2_, particularly during the later stage of the pyrolysis under high temperature (e.g., above 450 °C). In contrast, oil sample is pyrolyzed under anoxic condition during TSRP-GC-MS analysis, similar to when activation energy distribution was first introduced to the pyrolysis of coal (e.g., [35, 39]). This estimation is closer to the postulated conditions of the application of DAEM, which assumes the decomposition of complex organic material takes a large number of independent, parallel, first-order reactions with different activation energies reflecting variations in the bond strengths of species. Therefore, the calculated energy from TSRP-GC-MS, which does not involve the reaction with O_2_, measures more of the energy required to disrupt the matrix (the matrix strength) and to release the intact pyrolyzates (the enthalpy of vaporization).

## Results and discussion

### Changes in asphaltene through photooxidation

Photooxidation has been recognized as a key weathering process for spilled oil on oceanic surface water During the 2010 Deepwater Horizon oil spill, resulting in a rapid formation of polar or oxygenated hydrocarbons, or photo-generated asphaltenes [6]. Previous studies have explicitly demonstrated the great extent and rate of the incorporation of oxygen (e.g., up to 13% of oxygen by weight [6]) and rapid time scale (e.g., within 3 days [49]) of photooxidation. However, how the matrix of asphaltene, i.e., the binding of individual hydrocarbons within the matrix, changes before and after photooxidation remains unclear.

**Fig. 3:**
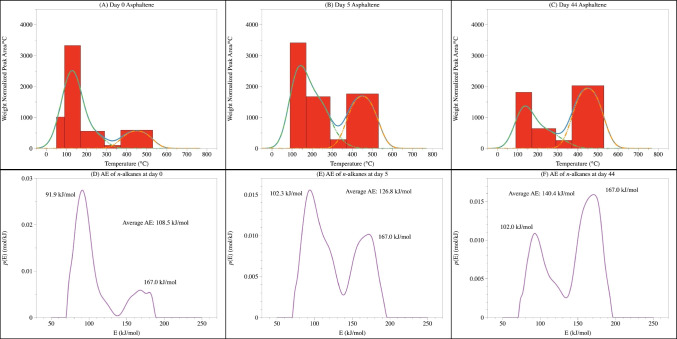
The intensities of aliphatic fractions in asphaltene at day 0 (**A**), day 5 (**B**), and day 44 (**C**), and their corresponding release energy distributions (**D**–**F**)

The pyrolysis data at different time points confirmed the prevalent presence of *n*-alkanes in asphaltene (Figure 3A to C), consistent with previous studies (e.g., FT-ICR MS [50, 51], pyrolysis GC-MS [19], and NMR analyses [25]). These *n*-alkanes are intact but embedded in the matrix, and the binding is strong enough to allow them to escape the organic solvent (e.g., hexane) during the asphaltene extraction.

**Fig. 4:**
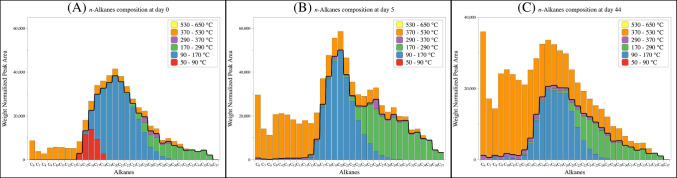
The molecular level composition of aliphatic fractions in asphaltene at day 0 (**A**), day 5 (**B**), and day 44 (**C**)

The distribution of these *n*-alkanes in asphaltene differed among different time points. At day 0, most *n*-alkanes (ca. 80%) were released within the low temperature thermal desorption zone (i.e., the sum of dehydration, decarboxylation, and depolymerization zones, equivalent to a temperature range of 50–370 °C), with only minor contribution from the high temperature cracking zone (370–650 °C, Figure 3A). As the photooxidation proceeded, the fraction of intact *n*-alkanes barely changed, but the *n*-alkanes generated from cracking significantly increased. By day 44, over 40% of total *n*-alkanes in the photo-generated asphaltene were from the cracking zone (Figure 3B, C). Molecular level identification of alkanes [19, 25] further revealed structural difference in the released *n*-alkane at different temperatures: the *n*-alkanes released in the cracking zone were dominated by shorter chain compounds (C_6_–C_16_) compared with the intact ones, but more long-chain alkanes were detected by day 44 (Figure 4).

The distribution pattern of *n*-alkanes corresponded to an average release energy of 108.5 kJ/mol for the alkane of day 0 asphaltene (Figure 3D). The intact alkanes released in the thermal desorption zone (50–370 °C) had an energy of 91.9 kJ/mol. The alkanes released in the thermal decomposition zone (370–650 °C) as the cracking product had a release energy of 167.0 kJ/mol, much higher than the regular H-bond [52], suggesting the involvement of breakdown of covalent bonds, i.e., these alkanes are covalently bonded to other hydrocarbon components. As the photooxidation process proceeded, the fraction of intact *n*-alkanes slightly decreased, but the *n*-alkanes generated from cracking significantly increased. By day 44, over 40% of total *n*-alkanes in the photo-generated asphaltene were from the cracking zone (Figure 3B, C), and the average release energy had increased from 108.5 kJ/mol at day 0 to 126.8 kJ/mol at day 5 and further to 140.4 kJ/mol at day 44 (Figure 3D to E). Such an increase was mainly driven by the increase of *n*-alkanes produced in the cracking zone. As previously mentioned, the calculated release energy in the thermal desorption zone represented the energy to disrupt intermolecular interactions with other molecules (i.e., the matrix strength) and the enthalpy of vaporization. Given an average enthalpy of vaporization for pure *n*-alkanes at ca. 69 kJ/mol (Table 2) [53–57], the matrix strength can therefore be estimated to be in the range of 20–30 kJ/mol. This estimated value is slightly higher than known intermolecular forces, such as electrostatic interaction (6–12 kJ/mol), H-bonding (4–13 kJ/mol), Van der Waals (dipole-dipole; 2–4 kJ/mol), and hydrophobic interaction (~5 kJ/mol), but within the range of stronger forces like Pi-Pi interaction (~40 kJ/mol). This further explained why *n*-alkanes in asphaltenes cannot be efficiently extracted to the saturated fraction during SARA fractionation, as solvent extraction is generally considered to be effective in the disruption of weak intermolecular forces (e.g., Van der Waals forces, less than 10 kJ/mol). The matrix strength of asphaltenes, therefore, is higher than that solvent extraction can provide (e.g., [58]).

**Fig. 5:**
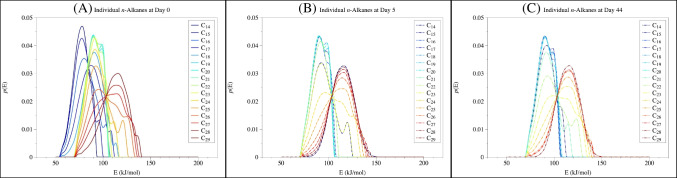
Release energy distribution of individual *n*-alkanes (C_14_–C_29_) from asphaltenes at day 0 (**A**), day 5 (**B**), and day 44 (**C**), with a clear shift to higher energy level in the averaged release energy

In addition to total *n*-alkanes, individual *n*-alkanes can be identified from TSRP-GC-MS (Table 2). Using *n*-alkanes with C numbers of 14–29 as examples, an overall pattern of increasing release energy with increasing C number can been seen in all three samples (Table 2, *R*^2^ = 0.91, *p* = 8.0 × 10^−9^ at day 0; *R*^2^ = 0.33, *p* = 0.02 at day 5; *R*^2^ = 0.87, *p* = 1.6 × 10^−7^ at day 44). To compare, previous studies showed that the enthalpies of vaporization of pure alkanes (up to C_30_) are generally in the range of ca. 54 kJ/mol for C_18_ to ca. 79 kJ/mol for C_29_ (average of 69 kJ/mol, Table 2 [53–57]). Consistent with the observed trend in total *n*-alkanes, similar increases in the release energy, the matrix strength, and the maximal releasing temperature were also observed in individual *n*-alkanes (Table 2). Specifically, release energy increased from 76.6–112.2 kJ/mol at day 0, to 90.3–114.6 kJ/mol at day 5, and finally to 90.4–114.6 kJ/mol at day 44 (*p* = 5.8 × 10^−6^; two-way ANOVA, Table 2), along with a clear shift to higher energy documented in the distribution of the release energy (Figure 5). Similarly, matrix strength (only for those *n*-alkanes with known enthalpies of vaporization) also increased from 21.7–35.5 kJ/mol at day 0, to 23.8–36.8 kJ/mol at day 5, and to 27.4–38.4 kJ/mol at day 44 (*p* = 0.001; two-way ANOVA, Table 2).

Such trend suggested that the photooxidation process affected the energetic behavior of asphaltenes, which were closely correlated with the changes in chemical structures. Notably, even the short-chain *n*-alkanes (e.g., C_14_) exhibited increasing release energy with proceeding photooxidation (Figure 5), indicating that the release of low molecular weight pyrolyzates was not governed solely by their inherent evaporation temperatures. Instead, the observed shift towards higher releasing temperature supported the role of matrix in maintaining the structure of photo-generated asphaltenes. This is the first quantitative data showing that photooxidation leads to an increase in the binding strength of individual *n*-alkanes within the asphaltene matrix. The structural modifications induced by photooxidation, such as the long alkyl side generated by the ring-opening reactions of aromatic structure [25], can lead to increased cross-linking among *n*-alkane-like molecules, which can result in a stronger matrix. These findings represent a significant step toward understanding how photooxidation alters the molecular environment of asphaltenes and highlight the importance of linking chemical structures within a sample matrix.

**Table 2:** Energy (kJ/mol) required to release different *n*-alkanes (C_14_ – C_29_) from asphaltenes at Day 0, 5, and 44, and the associated maximum release temperature (K)

*n*-Alkanes	Day 0		Day 5		Day 44		Enthalpy of vaporization (kJ/mol) *
	E (kJ/mol)	Temperature (K)	E (kJ/mol)	Temperature (K)	E (kJ/mol)	Temperature (K)	
C14	76.6 ± 8.0	341.2	114.6 ± 11.3	502.9	92.0 ± 9.2	405.8	
C15	78.3 ± 9.1	345.4	95.8 ± 12.1	423.1	90.7 ± 8.2	401.7	
C16	82.0 ± 10.8	354.2	90.9 ± 8.3	402.2	90.6 ± 8.1	401.0	
C17	86.5 ± 11.2	383.5	90.5 ± 8.0	400.8	90.4 ± 8.0	400.4	
C18	89.8 ± 10.4	397.8	90.3 ± 7.9	399.6	90.5 ± 8.0	400.8	54.4
C19	90.5 ± 8.0	400.4	90.5 ± 8.0	400.4	90.6 ± 8.1	401.1	56.0
C20	90.3 ± 7.9	400.0	90.3 ± 7.9	399.9	90.9 ± 8.3	402.5	
C21	90.3 ± 7.9	399.9	90.6 ± 8.1	400.9	93.5 ± 10.4	413.4	66.1
C22	90.6 ± 8.0	400.9	91.6 ± 8.8	405.3	98.8 ± 13.6	436.0	67.8
C23	91.2 ± 8.5	403.8	96.0 ± 12.2	423.8	104.3 ± 14.7	459.2	69.5
C24	92.8 ± 9.8	410.6	103.2 ± 14.7	454.3	108.4 ± 14.3	476.7	71.2
C25	96.5 ± 12.5	426.1	107.8 ± 14.5	473.8	111.2 ± 13.4	488.1	72.7
C26	102.3 ± 14.6	450.6	110.9 ± 13.5	487.3	113.1 ± 12.4	496.3	74.3
C27	106.0 ± 14.7	466.3	112.6 ± 12.7	494.1	113.4 ± 12.2	497.8	75.8
C28	108.8 ± 14.2	478.4	113.5 ± 12.1	498.3	114.6 ± 11.3	502.9	77.3
C29	112.2 ± 12.9	492.7	114.2 ± 11.6	501.1	114.6 ± 11.3	502.9	78.3

*The value here represents the enthalpy of vaporization at the normal boiling point for pure *n*-alkanes based on experimental measurements from literatures [53–57]

### Changes in the matrix strength of individual *n*-alkanes from crude oil to tarball

Changes in the composition of crude oil with weathering have been extensively studied. Various weathering processes, including evaporation [49, 59], dissolution [60], emulsification [61], biodegradation [62, 63], and abiotic chemical transformation [6, 64], all affect the composition and fate of crude oil. Previous studies, either using the traditional wet chemical analysis (e.g., [27]) or TSRP-GC-MS [19], have shown an overall depletion of low molecular weight *n*-alkanes with weathering [27]. However, there have been no studies to demonstrate the difference in the detected *n*-alkanes from different stages of weathered oil from the perspective of matrix strength or energy.

**Fig. 6:**
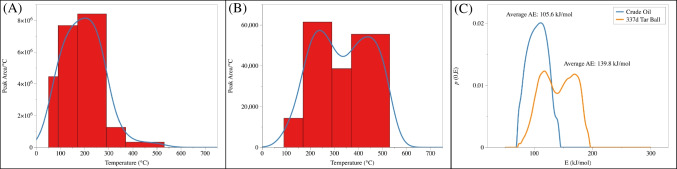
The distribution pattern of different total *n*-alkanes over the temperature range of 50 to 650 °C in crude oil (**A**) and 337d tarball (**B**)

Demonstrated here is the TSRP-GC-MS analysis of crude oil and tarball (337 days after the DWH spill) samples. The samples were pyrolyzed in 6 consecutive thermal slices (50–90 °C, 90–170 °C, 170–290 °C, 290–370 °C, 370–530 °C, and 530–650 °C), with the thermal desorption zone covering the temperature range of 50–370 °C and cracking zone covering 370–650 °C. Compared with the high temperature cracking zone, the *n*-alkanes released in the thermal desorption zone were considered as “intact” and thus represent the matrix better, since they are the sum of total non-covalent intermolecular interactions, of the pyrolyzed oil sample. The total amount of *n*-alkanes (C_9_–C_37_) occurred in the four lower thermal slices, or the thermal desorption zone (50–370 °C) dominated the thermogram of crude oil at a percentage of over 97% (Table 3). Specifically, the slice of 90–170 °C was responsible for 31% of total *n*-alkanes, while 290–370 ℃ held nearly 52% of total *n*-alkanes (Table 3 and Figure 6A). To compare, only 3% of *n*-alkanes were detected in the fragmentation zone (370–650 °C). On the other hand, in the 337 d tarball the percentage of intact *n*-alkanes has dropped to 57%, while 43% of the detected “alkanes” were produced above cracking temperature (≥ 370 °C, Table 3, Figure 6B).

Such differences in the *n*-alkane distribution have led to a correspondingly different activation energy distribution (Figure 6C). The averaged release energy of total *n*-alkanes in 337 d tarball (139.8 kJ/mol) was much higher than that in crude oil (105.6 kJ/mol). Even if only intact *n*-alkanes from thermal desorption zones were taken into consideration, the release energy in crude oil (104.4 kJ/mol) was still much lower than that in tarball (118.7 kJ/mol).

TSRP-GC-MS offers a unique approach to estimate the energy required to volatilize specific compounds from the complex nature mixture. Based on a comparison with *n*-alkane standards, the distribution of individual *n*-alkanes from C_14_ to C_29_ can be obtained. The pattern in bulk *n*-alkanes was also reflected in individual ones. For instance, 52% of C_14_ was detected in the temperature range of 90–170 °C in crude oil, with 18% and 30% in 50–90 °C and 170–290 °C, respectively. In contrast, 100% of C_14_ was detected from the slice of 290–370 °C in the 337 d tarball. This has led to a specific release energy of 99.9 kJ/mol for C_14_ in crude oil, but over 138.4 kJ/mol in tarball, suggesting that even though it was the same compound (Table 4), it was bonded more tightly in the tall bar than the crude oil.

**Fig. 7:**
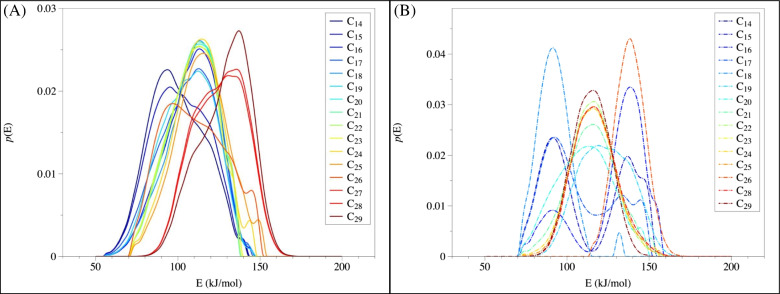
Release energy distribution of individual *n*-alkanes (C_14_–C_29_) in crude oil (**A**) and tarball (**B**), with a clear shift to higher energy level in the averaged release energy

**Fig. 8:**
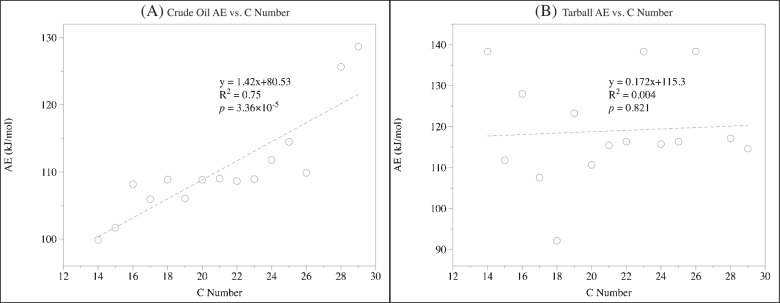
The correlation between release energy of specific *n*-alkanes vs. C number of *n*-alkanes in (**A**) crude oil and (**B**) tarball. A clear and significant positive correlation is observed for crude oil (*R*^2^ of 0.75 and *p* of 3.36 × 10^–5^), but not for tarball (*R*^2^ of 0.004 and *p* of 0.821)

The release energy of individual *n*-alkanes, which represents the matrix strength and the enthalpy of vaporization for the specific *n*-alkanes from the matrix, ranged from 99.9 to 128.7 kJ/mol in crude oil, but from 92.1 to 138.4 kJ/mol in tarball samples (Table 4, Figure 7). For *n*-alkanes with reported enthalpies of vaporization, an insignificant but generally increasing trend in matrix strength (*p* = 0.15, Table 4) was documented. Similar to the case of photo-generated asphaltenes (“Changes in asphaltene through photooxidation” section), the release energy generally increased as C number increased in crude oil samples (*R*^2^ = 0.75; *p* = 3.4 × 10^−5^, Figure 8A). However, no clear trend was observed in tarball (*R*^2^ = 0.004; *p* = 0.82 in tarball, Figure 8B). Individual *n*-alkanes were retained in the matrix of crude oil mainly through intermolecular forces, among which the hydrophobic forces (i.e., interactions among non-polar molecules) may have played an important role. The increase in C number of *n*-alkanes could result in an increasing hydrophobicity, which further increases the strength of hydrophobic forces and leads to a stronger matrix strength. The lack of clear trend in release energy with increasing C number in tarball, however, suggested a change in energetic behavior as release energy of different individual *n*-alkanes became more homogenized. After extensive weathering process [51, 64], the same *n*-alkanes that used to be retained in crude oil through non-polar reactions may now have been occluded in the tarball by totally different but much stronger interactions. To further support this interpretation, there was significant increase in release energy from crude oil to tarball (Table 4, *p* = 0.03; paired t-test). Generally, relatively low energy was required to disrupt these forces to release the individual *n*-alkanes from crude oil. Such increase in release energy in *n*-alkanes also reflected why these tarballs could be preserved in the environment for an extended period, as more energy is required to access to the *n*-alkanes biologically and abiotically.

**Table 3:** Intensities of total *n*-alkanes in each thermal slice for crude oil and tarball samples

Slices	Crude	337 d tarball
50–90 °C	178,554,716	0
90–170 °C	615,045,289	1,144,836
170–290 °C	1,008,686,137	7,380,979
290–370 °C	101,497,721	3,086,612
370–530 °C	52,420,346	8,879,880
530–650 °C	0	0

**Table 4:** Calculated energy (kJ/mol) required to release different *n*-alkanes (C_14_– C_29_) from crude oil and 337 d tarball

*n*-Alkanes	Crude oil	337 d tarball	Enthalpy of vaporization*
C14	99.9 ± 16.8	138.4 ± 9.1	
C15	101.7 ± 17.0	111.8 ± 24.6	
C16	108.2 ± 15.6	128.0 ± 21.2	
C17	106.0 ± 16.6	107.6 ± 21.1	
C18	108.9 ± 15.5	92.1 ± 10.4	54.4
C19	106.0 ± 16.1	123.3 ± 15.6	56.0
C20	108.8 ± 14.3	110.7 ± 16.7	
C21	109.0 ± 14.2	115.4 ± 15.2	66.1
C22	108.7 ± 14.4	116.3 ± 12.8	67.8
C23	108.9 ± 14.4	138.4 ± 9.1	69.5
C24	111.8 ± 14.8	115.7 ± 13.6	71.2
C25	114.5 ± 16.0	116.3 ± 13.3	72.7
C26	109.9 ± 19.0	138.4 ± 9.1	74.3
C28	125.7 ± 15.7	117.1 ± 13.3	75.8
C29	128.7 ± 15.4	114.6 ± 11.3	77.3

*The value here represents the enthalpy of vaporization at the normal boiling point for pure *n*-alkanes based on experimental measurements from literatures [53–57]

## Comments and recommendations

This work demonstrated that TSRP-GC-MS results provided insights into the molecular structure as well as the possible formation pathway of photo-generated asphaltene. Furthermore, by elucidating the energy required to release intact *n*-alkanes, this analytical approach provided insights into the energetic characteristics of crude oil and tarballs. While it is a reasonable concern that the release energy estimated from TSRP-GC-MS may only reflect the evolved volatile fractions and therefore cannot connect the pyrolyzates with the bulk organic samples, results from this work suggested otherwise. The release patterns of *n*-alkanes differed in samples with different photooxidation degrees, showing that interactions with other organic compounds (i.e., matrix strength) played a key role in the volatilization of pyrolyzates. In addition, the identification of these pyrolyzates can reveal the key structure of the bulk sample. For instance, the detection of long-chain alkanes and alkenes in photo-generated asphaltenes via TSRP-GC-MS is consistent with the presence of long alkyl side chains [12]. Overall, TSRP-GC-MS provides important structural and energetic information on the complex petroleum-related organic matter.

Beyond understanding the molecular structure of petroleum-related samples, the application of DAEM in the interpretation of TSRP-GC-MS data can offer quantitative insights into the matrix that holds the numerous individual molecules. This technique potentially can be applied to other types of natural organic matter (NOM) samples, as the distribution pattern of different pyrolyzates released at different temperatures can be similarly transformed into the release energy that quantitatively describes the strength of the organic matrix, connecting the molecular structure of NOM and its behavior in the environment. This approach may be particularly useful in analyzing sedimentary organic matter, and sinking or suspended particles in aquatic environments, but can be easily expanded to dissolved components given the appropriate extraction method (e.g., solid-phase extraction and/or ultrafiltration; [29]). It also has the potential to be expanded as a tool for molecular level characterization of NOM. A pyrolyzates database, which is currently being constructed, can be built based on known biomolecules, and the structures of NOM can be computed through comparison with the database. This information could be key in furthering our understanding of the diagenesis and preservation of natural organic matter in the environment.

## Supplementary Information

Below is the link to the electronic supplementary material.

Supplementary Material 1 (DOCX 241 KB)

## Data Availability

Raw data of this work can be found in published work [19, 25]. The re-analysis of the raw data with specific focus on release energy can be found in Figshare (10.6084/m9.figshare.29987992.v1).
