# Supplementary material for: Deciphering the binding strength of oil matrix through molecularly resolved release energy analysis using thermal slicing ramped pyrolysis GC-MS
Source: Anal Bioanal Chem. 2025 Oct 10;417(28):6339–52. doi: 10.1007/s00216-025-06110-9 (PMC12598681; doi:10.1007/s00216-025-06110-9)
Supplement: Supplementary file 1 — Supplementary Material 1 (DOCX 241 KB) [file 216_2025_6110_MOESM1_ESM.docx]

**Deciphering the Binding Strength of Oil Matrix through Molecularly Resolved Release Energy Analysis Using Thermal Slicing Ramped Pyrolysis GC-MS**

Supplementary Materials

Kaijun Lu^*^ ^†^, Jianhong Xue^*^, and Zhanfei Liu^*^

^*^The University of Texas at Austin, Marine Science Institute 750 Channel View Dr., Port Aransas, TX 78373

^†^Coastal Carolina University, Department of Marine Science, 100 Chanticleer Drive East, Conway, SC 29526

Corresponding: [klu@coastal.edu](mailto:klu@coastal.edu)

**The calculation of activation energy**

As mentioned in the paper, the concept of an activation energy distribution was originally proposed by Vand [1] to investigate how the electrical resistance of metallic films changes, and was later applied to the study of the kinetics of volatile products from coal by Pitt [2] and Anthony and Howard [3]. Distributed activation energy model (DAEM) has now been widely applied in the pyrolysis field to probe the relationship between the thermal activation energy of organic matter and its weight change or the production of volatile matters (e.g., [4–6]). A more detailed description on the deducing process of activation energy based on DAEM is provided here, but has also been thoroughly discussed in many other works (e.g., [7–9]). The discrete DAEM assumes that each complex organic matter sample has a total of *i* chemical groups, and the contribution of the *i*th group to the overall production of volatiles of the whole sample following an *n*th order reaction:

|  | $\frac{d\left( {V_{i}}/{V_{i}^{*}} \right)}{dt}=k_{i}\left( \frac{V_{i}^{*}-V_{i}}{V_{i}^{*}} \right)^{n}$ | (1) |
| --- | --- | --- |

where *V_i_* is released mass fraction of group *i* at time *t*, *V_i_^*^* is the total released mass fraction of the *i*th group, *n* is the reaction order, and *k_i_* is the reaction constant of chemical group *i*. Because the reaction constant *k_i_* is related to temperature and activation energy, it can be further rewritten as a time-dependent function of activation energy *E_i_* following the Arrhenius equation:

|  | $k_{i}=A_{i}\exp\left( -\frac{E_{i}}{RT} \right)$ | (2) |
| --- | --- | --- |

in which *A_i_* is the empirically derived Arrhenius pre-exponential factor, or “frequency factor”, *R* is the ideal gas constant, and *T* is the measured temperature at time *t*.

The pyrolysis of an organic sample, either under anoxic condition or oxic condition, is assumed to follow first order reaction (i.e., *n* = 1; [4, 9]), so Eq. 1 can then be written in its integral form:

|  | $\frac{V_{i}}{V_{i}^{*}}=1-\exp\left[ -\int_{0}^{t} A_{i}\exp\left( -\frac{E_{i}}{RT} \right)dt \right]$ | (3) |
| --- | --- | --- |

From the perspective of the sample, the total amount of released volatile products would be the sum of all *i* chemical groups. The issues with this discrete model are that the total number of chemical groups (*i*) has to be set as a priori empirically, and that the model is prone to noise [9]. Instead, a continuous model, which assumes an inﬁnite number of irreversible ﬁrst order parallel reactions with continuously varying activation energies to occur simultaneously, may better describe the actual situation. Therefore, to replace the fixed *E_i_* of each chemical group with an *E* that varies continuously, the amount of volatile material can be written as:

|  | $dV^{*}=V^{*}f\left( E \right)dE$ | (4) |
| --- | --- | --- |

in which *V^*^* is the total amount of volatile products, d*V^*^* is the amount of volatile products at specific activation energy level of *E*, and *f(E)* is a distribution function that describes the probability for the chemical groups to have an activation energy of *E*. Deciphering *f(E)* thus becomes the focus of the problem.

Combine Eqs. (3) and (4), and replace the discrete component *V_i_* and *V_i_^*^* with their continuous counterparts of d*V*, which is the amount of volatile products at specific activation energy level of *E* at given time *t*, and d*V^*^*, respectively, to derive Eq. (5):

|  | $\frac{dV}{dV^{*}}=1-\exp\left[ -\int_{0}^{t} A\exp\left( -\frac{E}{RT} \right)dt \right]$ | (5) |
| --- | --- | --- |

|  | $dV=dV^{*}\left\{ 1-\exp\left[ -\int_{0}^{t} A\exp\left( -\frac{E}{RT} \right)dt \right] \right\}=V^{*}\left\{ 1-\exp\left[ -\int_{0}^{t} A\exp\left( -\frac{E}{RT} \right)dt \right] \right\}f\left( E \right)dE$ | (6) |
| --- | --- | --- |

The conversion factor *α*, which describes how much of the total amount of volatile products (*V^*^*) has already been released by time *t*, can be calculated by the integration of Eq. (6):

|  | $\alpha=\frac{V}{V^{*}}=1-\int_{0}^{\infty} \exp\left[ -\int_{0}^{t} A\exp\left( -\frac{E}{RT} \right)dt \right]f\left( E \right)dE$ | (7) |
| --- | --- | --- |

Given that the pyrolysis of organic matter is conducted at temperatures linearly increasing with time from a starting temperature *T_0_* and a ramping rate of *β*, Eq. (7) can be rewritten in terms of temperature *T*:

|  | $\alpha=\frac{V}{V^{*}}=1-\int_{0}^{\infty} \exp\left[ -\int_{T_{0}}^{T} \frac{A}{\beta}\exp\left( -\frac{E}{RT} \right)dT \right]f\left( E \right)dE=1-\int_{0}^{\infty} \Phi\left( E,T \right)f\left( E \right)dE$ | (8) |
| --- | --- | --- |

This classic equation of *α* has been frequently mentioned in different forms in the field of thermal analyses (e.g., [7–11]). During a thermogravimetric or pyrolysis process, the ramping rate *β* is often preset for a specific heating profile, and *α* is generally monitored as the amount of generated volatile products, or the amount of remaining mass of a sample (i.e., 1 - *α*). As the frequency factor *A* is also interrelated with *E* and *f(E)* (e.g., [10–12]), knowing how *α* changes with *T* at a given *β* would theoretically help to calculate the *f(E)* and understand the distribution function of activation energy.

Previous studies have taken various approaches to simplify Eq. (8) and to solve *A* and *f(E)*, including but not limited to assuming *A* as a constant [9], setting a priori for the distribution function of *f(E)* (e.g., Gaussian distribution [7], logistic distribution [13, 14], gamma distribution [15]), as well as the applications of various optimization methods. Miura and Maki [11] proposed an integral method to determine *A* and *f(E)* without any previous assumptions. The critical steps of this integral method are the simplifications of Eq. (8) with approximations, which are the Coats-Redfern approximation of Φ*(E, T)*, and the subsequent approximation of Φ*(E, T)* by a step function [7, 10, 11]. Such integral method has allowed an estimation of *A*, *E,* and *f(E)* based on the linear relationship between *1/T* and *β/T^2^*, which can be easily solved with only three pyrolysis experiments with different values of *β*. However, it has been pointed out that the oversimplification of Φ*(E, T)* by a step function might have resulted in significant errors [7, 9].

The most current approach to estimate the distribution function *f(E)* is provided by Hemingway et al. [9]. The core of this work was to use the inverse solution to calculate the *f(E)* that provides the best match of the calculated distribution of *α* vs. *T* with the observed distribution, with *A* set as a priori (i.e., a constant of 10^10^ s^-1^). A Tikhonov regularization factor *λ* is further introduced to minimize the issue of noises in measured data and to avoid an overfit. The extensive calculation is achieved with Python code and is accessible through the “rampedpyrox” package [9].

**Figures**

Figure S1. The derivative thermogravimetric (DTG) curve obtained from thermogravimetric analysis (TGA; blue) and the results from evolved gas analysis data (EGA; red and green), which is essentially the same as TSRP-GC-MS. The DTG from TGA shared a very similar pattern with that from EGA, given the similar condition (e.g., same temperature range to 650 ℃; anoxic condition under N_2_).

1. Vand V (1943) A theory of the irreversible electrical resistance changes of metallic films evaporated in vacuum. Proc Phys Soc 55:222. https://doi.org/10.1088/0959-5309/55/3/308

2. Pitt GJ (1962) The kinetic of the evolution of volatile products from coal. Fuel 41:267–274

3. Anthony DB, Howard JB (1976) Coal devolatilization and hydrogastification. AIChE Journal 22:625–656. https://doi.org/10.1002/aic.690220403

4. Cai J, Wu W, Liu R, Huber GW (2013) A distributed activation energy model for the pyrolysis of lignocellulosic biomass. Green Chem 15:1331–1340. https://doi.org/10.1039/C3GC36958G

5. Yang Y, Jiang M, Song L, Shen Y, Lei T, Cai J (2024) Systematical analysis and application of distributed activation energy model (DAEM) with Weibull distribution for pyrolysis kinetics of lignocellulosic biomass. Renewable Energy 237:121549. https://doi.org/10.1016/j.renene.2024.121549

6. Bach Q-V, Tran K-Q, Skreiberg Ø (2017) Combustion kinetics of wet-torrefied forest residues using the distributed activation energy model (DAEM). Applied Energy 185:1059–1066. https://doi.org/10.1016/j.apenergy.2016.02.056

7. Cai J, Li T, Liu R (2011) A critical study of the Miura–Maki integral method for the estimation of the kinetic parameters of the distributed activation energy model. Bioresource Technology 102:3894–3899. https://doi.org/10.1016/j.biortech.2010.11.110

8. Cai J, Wu W, Liu R (2014) An overview of distributed activation energy model and its application in the pyrolysis of lignocellulosic biomass. Renewable and Sustainable Energy Reviews 36:236–246. https://doi.org/10.1016/j.rser.2014.04.052

9. Hemingway JD, Rothman DH, Rosengard SZ, Galy VV (2017) An inverse method to relate organic carbon reactivity to isotope composition from serial oxidation. Biogeosciences 14:5099–5114. https://doi.org/10.5194/bg-14-5099-2017

10. Miura K (1995) A New and Simple Method to Estimate f(E) and k0(E) in the Distributed Activation Energy Model from Three Sets of Experimental Data. Energy Fuels 9:302–307. https://doi.org/10.1021/ef00050a014

11. Miura K, Maki T (1998) A Simple Method for Estimating f(E) and k0(E) in the Distributed Activation Energy Model. Energy Fuels 12:864–869. https://doi.org/10.1021/ef970212q

12. Du Z, Sarofim AF, Longwell JP (1990) Activation energy distribution in temperature-programmed desorption: modeling and application to the soot oxygen system. Energy Fuels 4:296–302. https://doi.org/10.1021/ef00021a014

13. Cai J, Jin C, Yang S, Chen Y (2011) Logistic distributed activation energy model – Part 1: Derivation and numerical parametric study. Bioresource Technology 102:1556–1561. https://doi.org/10.1016/j.biortech.2010.08.079

14. Cai J, Yang S, Li T (2011) Logistic distributed activation energy model – Part 2: Application to cellulose pyrolysis. Bioresource Technology 102:3642–3644. https://doi.org/10.1016/j.biortech.2010.11.073

15. Burnham AK, Braun RL (1999) Global Kinetic Analysis of Complex Materials. Energy Fuels 13:1–22. https://doi.org/10.1021/ef9800765
